# Prediction of postoperative facial swelling, pain and trismus following third molar surgery based on preoperative variables

**DOI:** 10.4317/medoral.18039

**Published:** 2012-12-10

**Authors:** Thiago de Santana-Santos, Jadson A. de Souza-Santos, Paulo R. Martins-Filho, Luiz C. da Silva, Emanuel D. de Oliveira e Silva, Ana C. Gomes

**Affiliations:** 1 Pernambuco Dental School (FOP/UPE), Camaragibe, Pernambuco, Brazil; 2 Ribeirão Preto Dental School (FORP/USP), Ribeirão Preto, São Paulo, Brazil; 3 Federal University of Sergipe (UFS), Aracaju, Sergipe, Brazil

## Abstract

Objective: This paper investigates the relationship between preoperative findings and short-term outcome in third molar surgery. Study design: A prospective study was carried out involving 80 patients who required 160 surgical extractions of impacted mandibular third molars between January 2009 and December 2010. All extractions were performed under local anesthesia by the same dental surgeon. Swelling and maximal inter-incisor distance were measured at 48 h and on the 7th day postoperatively. Mean visual analogue pain scores were determined at four different time periods. Results: One-hundred eight (67.5%) of the 160 extractions were performed on male subjects and 52 (32.5%) were performed on female subjects. Median age was 22.46 years. The amount of facial swelling varied depending on gender and operating time. Trismus varied depending on gender, operating time and tooth sectioning. The influence of age, gender and operating time varied depending on the pain evaluation period (p < 0.05). Conclusions: Short-term outcomes of third molar operations (swelling, trismus and pain) differ depending on the patients’ characteristics (age, gender and body mass index). Moreover, surgery characteristics such as operating time and tooth sectioning were also associated with postoperative variables.

** Key words:**Third molar extraction, pain, swelling, trismus, postoperative findings, prediction.

## Introduction

The difficulty in assessing impacted third molars is perhaps the most important individual factor to consider in referring such cases to specialists (1). A number of efforts at determining a model for this assessment have been made, but none can be said to be universally reliable. The first attempt to create a model of this nature was published by MacGregor (2) in 1979, who established a multivariate model based on panoramic radiographic findings.

The surgical removal of third molars (wisdom teeth) generally produces pain, trismus and facial swelling in the postoperative period (3). The many factors that contribute toward these conditions are complex, but originate in an inflammatory process initiated by surgical trauma (4). The postoperative effect of wisdom tooth surgery on quality of life is reported to be threefold greater in patients who experience pain, swelling and trismus (either alone or in combination) in comparison to asymptomatic patients (5). Many clinicians have therefore stressed the need for better pain, swelling and trismus control in patients who undergo third molar surgery (6). There have been few attempts to study patients’ expectations regarding outcomes, although patients’ perceptions of recovery following third molar surgery have been reported (5,7-9).

Winter’s (10), Pell and Gregory’s (10), Pederson’s (11) and the WHARFE (Winter’s classification, Height of the mandible, Angulation of second molar, Root shape and morphology, Follicle development, Exit path) (12) classification/scoring systems figure prominently among the models proposed. These early systems employed quantitative scores for each parameter and difficulty was estimated based on the total radiographic score of an impacted tooth. Such attempts were based exclusively on radio-graphic variables (2,13), whereas recent evidence has associated a wide variety of non-radiographic variables with difficulty during impacted third molars extractions (14-17). However, the magnitude of the contributions of the different categories of variables remains to be quantified.

While recent literature may offer further information for a better estimation of the difficulty of third molar surgery, the findings are conflicting and there is considerable variation in the factors involved. A systematic review of the literature carried out to identify important variables that have been consistently listed as determinants of surgical difficulty (determined based on operating time) reports the most current evidence applicable to clinical practice in relation to the evaluation and surgical management of impacted mandibular third molars. The most consistent determinants of difficulty are age, surgical procedure, number of teeth extracted, depth angle and root morphology (1).

Berge and Bøe (18) attempted to predict the extent of postoperative morbidity through multiple regression analysis. However, the study did not correlate the extent of postoperative facial swelling and pain with preoperative variables, but rather with overall predictive factors. Yuasa and Sugiura (19) and Barbosa-Rebellato et al. (20) found that swelling and pain differ depending on the characteristics of the patient (age and gender) and preoperative difficulty index. It is more informative from the patient’s point of view to relate outcome to factors that can be measured preoperatively than to rely on an overall probability (19).

The aim of the present paper was to investigate the correlation between preoperative factors (age, gender and body mass index), intraoperative factors (surgical duration and tooth sectioning) and postoperative morbidity (short-term outcome: swelling, trismus and pain) in the extraction of third molars.

## Material and Methods

The present study received approval from the Institutional Ethics Committee (CEP/UPE: 101/09). The subjects were selected from a pool of patients admitted for dental treatment between January 2009 and December 2010. All participants signed a statement of informed consent.

A prospective study was carried out involving 80 healthy, non-smoking patients scheduled for the bilateral surgical removal of symmetrically placed impacted lower third molars. The subjects had no known immune impairment, no contraindications for oral surgery and were not taking any medication. Orthopantomographic radiograms were taken to ensure the similarity of the tooth inclinations based on Winter’s classification (only vertical and mesioangular positions were used) and the Pell & Gregory classification (only class B and position 1 were used) (10).

A single examiner performed all clinical measurements prior to surgery (baseline) as well as at 48 h and on the seventh day in the postoperative period. Swelling measurements were taken using a 2-0 nylon thread and a millimeter ruler. Trismus measurements were taken using a calibrated digital caliper. To evaluate swelling, markings with permanent marker were made prior to surgery on the following facial regions: angle of the mandible, tragus, labial commissure, nasal border, soft pogonion and laterally to the outer corner of the eye. Five distance measurements (I to V) were taken (10). Trismus was evaluated by measuring the distance between the incisal edges of the upper and lower central incisors. Pain intensity was assessed using a 10-level visual analog scale (VAS) with the patient placing a mark on the scale to indicate an intensity range from no pain [0] to severe/unbearable pain [10] (10). Pain was evaluated 4 h, 12 h, 24 h and 48 h postoperatively. The amount of the analgesia (acetaminophen 750 mg) taken in the postoperative period was also recorded.

The preoperative treatment protocol for all patients included the prescription of 8 mg of dexamethasone and 1 g of amoxicillin taken orally one hour prior to surgery. The surgeon ensured that all patients knew how to take the prescribed medication.

Each patient was operated by the same senior oral and maxillofacial surgeon, using the same surgical technique on both sides in order to minimize discrepancies in the handling of oral tissues. Extra-oral antisepsis was performed with a 2.0% chlorhexidine solution and intraoral antisepsis was performed with a 0.12% chlorhexidine rinse. Blocking of the inferior alveolar, lingual and buccal nerves was carried out using 2.0% lidocaine with 1:100,000 epinephrine. To perform the surgical procedure, materials and instruments routinely required for this surgery were used and the standardized technique was performed. Briefly, an “L” shaped incision was made and a mucoperiosteal flap was raised. When osteotomy and tooth sectioning were performed on one side, the other side received the same treatment in order to standardize the surgical trauma. All procedures were performed under abundant irrigation with sterilized 0.9% physiological solution. The closure of the mucoperiosteal flap was performed with 3-0 silk. The difficulty of the removal procedure was determined based on the four grades proposed in Campbell’s method: (I) simple tooth extraction; (II) bone removal or tooth division; (III) bone removal and tooth division; and (IV) the same as III, but very difficult. Grade II and III surgeries were considered in the present study (10). The duration of the surgical procedure was counted from incision until tooth removal. One impacted lower third molar was removed on the first surgical visit and the contralateral lower third molar was removed on the second surgical visit, which was scheduled for three weeks later.

On the first day following surgery, the patients were authorized to take analgesics (acetaminophen 750 mg four times daily) only in case of pain. Acetaminophen was also used as the rescue drug. The patients were instructed to eat only soft food and to abstain from mouth washing for the first 24 h and from brushing and flossing around the surgical area until suture removal (14 days following surgery). For plaque control, the patients used a 0.12% chlorhexidine mouth rinse for one minute twice a day for two weeks postoperatively.

The patients were required by protocol to return for follow-up 48 h and seven days following surgery. All postoperative data were recorded by the same independent blinded investigator in order to avoid observer bias. At each appointment, the presence of paresthesia, fatigue or infectious complications was noted and the patients were examined with regard to the main variables (swelling, trismus and pain).

The following variables were recorded before surgery: age (continuous and categorical variables: 15 to 20 and 21 to 30 years); gender (male, female); body mass index (BMI) (continuous and categorical: < 18.5, 18.6 to 24.99, 25.0 to 29.99 and over 30); and facial measurements (five distance measurements).

Facial swelling was calculated by the sum of the five measurements divided by five and percentage of facial swelling was calculated as preoperative measurement minus postoperative measurement divided by preoperative measurement times 100. These measurements and those of maximal interincisal distance (MID) were made on postoperative days 2 and 7 by the same person. Trismus was calculated as preoperative measurement minus postoperative measurement divided by preoperative measurement multiplied by 100.

Differences in mean swelling, trismus and pain in relation to gender, age and tooth sectioning were analyzed using the Mann-Whitney U test. ANOVA followed by the Bonferrroni test was used for comparisons of mean swelling and trismus according to body mass index and duration of surgery. The Kruskal-Wallis test followed by Dunn’s post hoc test was used for the comparison of mean swelling and trismus according to body mass index and duration of surgery. Spearman’s correlation coefficients were calculated to determine the relation between duration of surgery and mean pain. All calculations were made using the OriginPro 8.0 statistical software program (OriginLab Corporation, Northampton, MA, USA). For all tests, a probability of less than 0.05 was considered significant.

## Results

Ninety-two individuals participated in the present study. However, 12 were excluded from the sample for not meeting the eligibility criteria. A total of 108 (67.5%) of the 160 remaining extractions (80 patients) were performed on males and 52 (32.5%) were performed on females [mean age ± standard deviation: 22.46 ± 4.11 years (range: 15 to 30 years). All clinical indications for removal were impaction (100%).

The statistical analysis revealed that the factors that predicted swelling on Day 2 were gender and operating time (p < 0.05). Those that predicted trismus were gender, operating time and tooth sectioning (p < 0.05). On Day 7, the only factor predictive of continued swelling and trismus was gender ( Table 1).

**Table 1:**
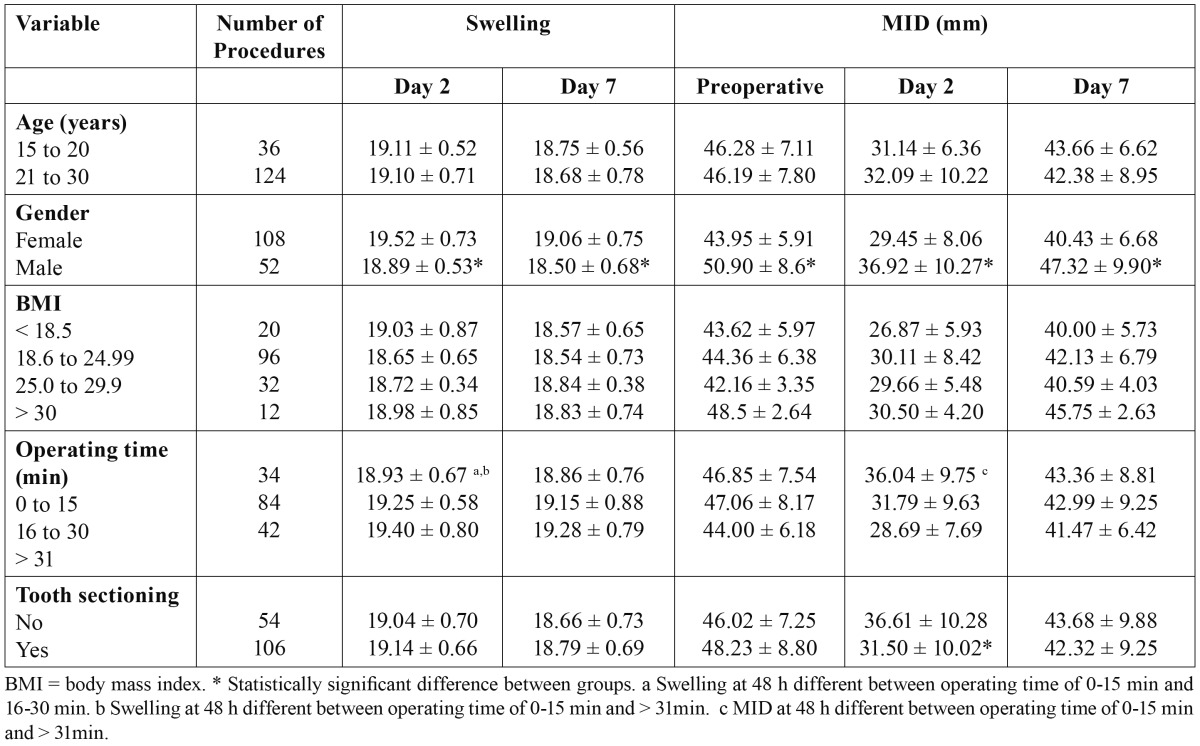
Mean swelling and maximal inter-incisal distance (MID) in relation to independent variables.

Regarding the mean VAS score, a younger age (15 to 20 years), the female gender, operating time > 31 min and tooth sectioning were predictive of greater pain at 4 and 12 h (p < 0.05). At 24 and 48 h, operating time alone was predictive of greater pain ( Table 2). The correlation between operating time and mean VAS score in the first 48 hours was moderate (Spearman correlation coeffi-cient: rs = 0.4; p < 0.0001). Considering the outcome factors together as a measure of morbidity, operating time was the predictive factor of swelling, trismus and pain on Day 2 (p < 0.05).

**Table 2:**
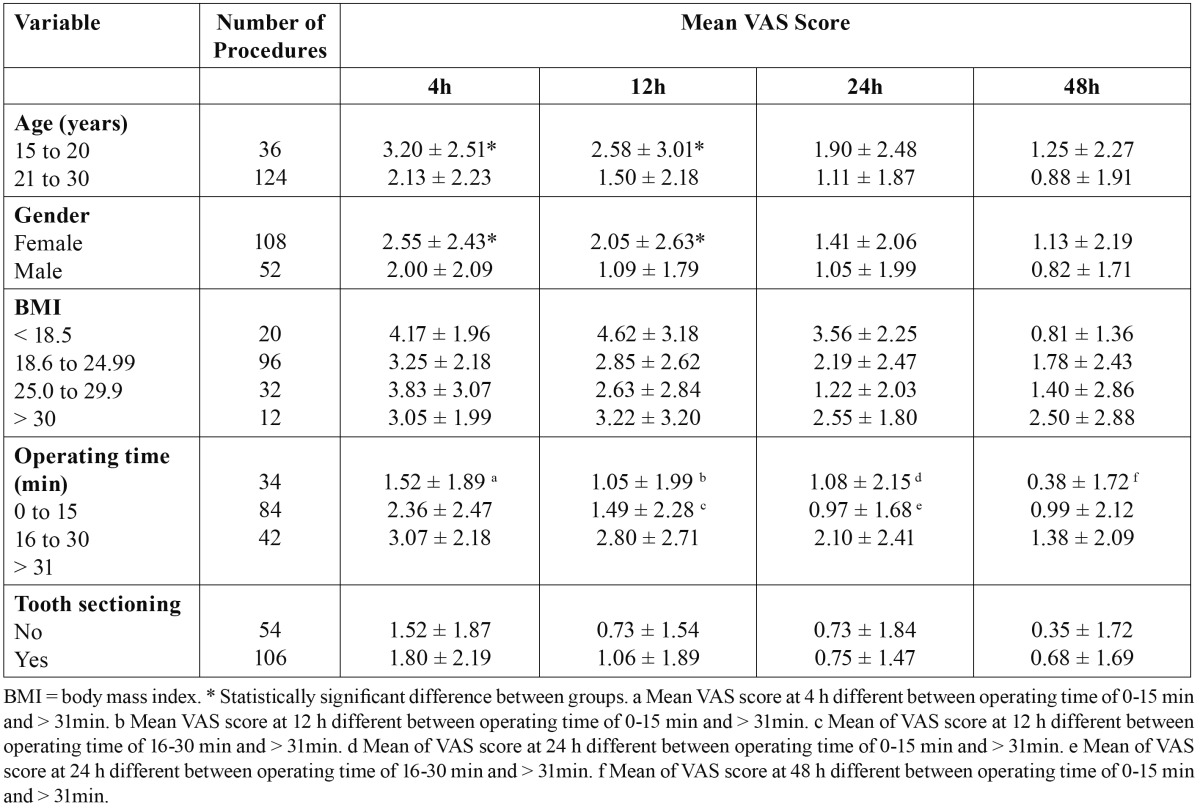
Mean visual analog scale (VAS) score in relation to independent variables.

## Discussion

The extraction of mandibular third molars is one of the most common surgical events (16). Thus, despite the diversified demands of practice, dental surgeons still face the problem of the removal of impacted mandibular third molars (21). Both the patient and dentist must therefore have scientific evidence-based information concerning the estimated degree of surgical difficulty in each case (22).

MacGregor (2) made the first attempt to establish a model for assessing surgical difficulty. The classic Pell and Gregory classification has recently been found to be inadequate for the determination of surgical difficulty (23). There are a number of previous studies carried out to evaluate surgical difficulty in the extraction of impacted mandibular third molars (13,14,16,17,19,21,23). However, most of these studies are only based on dental factors determined through radiologic assessments (13,16,17,19,23). While opinions may vary, most authors agree that these radiologic factors play some role in estimating difficulty (13,14,16,17,23). Other authors believe it is difficult to estimate difficulty through radiologic methods alone and that actual difficulty can only be estimated intraoperatively (24). Some authors also believe that clinical variables, such as patient age, gender and weight, are also very important (14,16). Few authors have proposed indexes for measuring surgical difficulty (17,19). Pederson proposed such an index (25), but it is seldom used due to reports that it does not match actual surgical difficulty (11,17).

Moreover, few studies (18,19) have attempted to predict the extent of postoperative morbidity using preoperative and intraoperative characteristics rather than the assessment of surgical difficulty. It should be stressed that that a risk factor that leads to greater surgical difficulty also increases the extent of the postoperative morbidity. In addition, discontent and litigation among patients is a problem caused by frequent complaints as pain (either during treatment or afterwards), major swelling, disturbances of trigeminal or facial nerve function, poor scar formation, and discrepancies between the expected and the actual result of treatment. However, a considerable proportion is practical consequences of the operation; the patients in turn tend not to ask about possible complications (26).

The female-to-male gender proportion in the present study was almost 1:2, which is in disagreement with a previous study reporting that women seek third molar surgery more frequently than men (12). The female gender is a risk factor due to the lesser bone thickness of the mandible (27). In the present study, gender was a determinant of greater morbidity in the postoperative period, which corroborates findings described by Benediktsdóttir et al.(14), Blondeau and Daniel (15) and Yuasa and Sugiura (19) and is in disagreement with findings described by Barbosa-Rebellato et al. (20) and Carvalho and do Egito Vasconcelos (12).

According to a number of authors, age is the most consistent factor in the determination of surgical difficulty (1,20). In the present study, this variable was only a predictive factor for pain variation in the postoperative period. Age is commonly reported to be significant to the occurrence of complications (14-16,19,22). The positive correlation may be related to the increase in bone density, which may require more handling during the operation. Moreover, an increase in age is associated with complete root formation, which may be related to the higher rate of complications among patients over 25 years of age (12). Few complications occurred in the present study, as only two patients experienced postoperative infection, which is in agreement with findings re-ported by Yuasa and Sugiura (19).

The amount of facial swelling varied depending on gender and operating time. Trismus varied depending on gender, operating time and tooth sectioning. Differences in mean VAS scores were associated with age, gender and operating time. It is quite likely that facial swelling is affected by individual characteristics, such as age. A previous study reports such a result in the univariate analysis, with gender as a predictive factor for facial swelling as well (19).

A total of 27.5% percent of the sample was overweight (BMI > 25 kg/m2). Surgical difficulty in such cases is attributed to the projection of the cheek tissue (12). However, no significant association was found in the present study to confirm this factor as predictive of swelling, trismus or pain. On the other hand, a lesser BMI value (< 18.5) characterizing underweight individuals was found in 13.75% of the sample, for whom VAS scores were higher at 4, 12 and 24 h. This finding may be attributed to secondary postoperative hypersensitivity, which may be exacerbated in underweight individuals. However, this issue needs to be investigated in further studies.

A number of studies have used operating time and surgical technique as determinants of difficulty (1,20,22,28). In one study, the authors found both these factors to be reliable, statistically significant measures and the best way to predict surgical difficulty (28). All procedures analyzed in the present study were categorized as having a moderate degree of difficulty and the surgical technique most often used for the removal of lower third molars was ostectomy in the company of tooth sectioning, with a mean operating time was 20.18 minutes. The correlation between operating time and mean VAS score in the first 48 hours was moderate (rs = 0.4). Surgical difficulty was standardized in order to increase the probability of similar surgical trauma, which allowed a better comparison of predictive variables and independent variables.

Further studies are needed to confirm the predictive factors described in this paper. To enhance the statistical analysis of the present study and minimize bias, the methodology employed was different from that reported in similar previous studies (18,19) in terms of being more specific with age limitations (15 to 30 years), vertical and mesioangular positions (Winter’s classification), Pell and Gregory Class I-B and Campbell grades II and III.

Based on the findings of the present study, short-term outcomes of third molar surgery (swelling, trismus and pain) differed depending on the characteristics of the patient (age, gender and body mass index). Surgery characteristics, such as operating time and tooth sectioning, were also associated with postoperative variables. However, due to the observational nature of the study, the results should be interpreted with caution. Further studies should be carried out on associations between preoperative findings and short-term outcomes of third molar extractions.
